# Supplementary material for: HIVprotI: an integrated web based platform for prediction and design of HIV proteins inhibitors
Source: J Cheminform. 2018 Mar 9;10:12. doi: 10.1186/s13321-018-0266-y (PMC5845081; doi:10.1186/s13321-018-0266-y)
Supplement: Supplementary file 2 — Additional file 2. Source code of HIVProtI web server. [file 13321_2018_266_MOESM2_ESM.zip › HIVprotI_Source-code/hivprotI/weka-3-6-6/doc/weka/experiment/InstanceQuery.html]

InstanceQuery


---


|  |  |  |
| --- | --- | --- |
| **Overview** \| **Package** \| **Class** \| **Tree** \| **Deprecated** \| **Index** \| **Help** \| **Weka's home** | |  |
| **PREV CLASS**   **NEXT CLASS** | **FRAMES**    **NO FRAMES**     **All Classes** |  |
| SUMMARY: NESTED \| FIELD \| CONSTR \| METHOD | DETAIL: FIELD \| CONSTR \| METHOD |  |


---


## weka.experiment Class InstanceQuery

```
java.lang.Object
  weka.experiment.DatabaseUtils
      weka.experiment.InstanceQuery
```

**All Implemented Interfaces:**: java.io.Serializable, OptionHandler, RevisionHandler

---

``` public class InstanceQuery extends DatabaseUtils implements OptionHandler ```

Convert the results of a database query into instances. The jdbc
driver and database to be used default to "jdbc.idbDriver" and
"jdbc:idb=experiments.prp". These may be changed by creating
a java properties file called DatabaseUtils.props in user.home or
the current directory. eg:

```` ```
 jdbcDriver=jdbc.idbDriver
 jdbcURL=jdbc:idb=experiments.prp
``` ```` 

Command line use just outputs the instances to System.out.


Valid options are:

```
 -Q <query>
  SQL query to execute.
```

```
 -S
  Return sparse rather than normal instances.
```

```
 -U <username>
  The username to use for connecting.
```

```
 -P <password>
  The password to use for connecting.
```

```
 -D
  Enables debug output.
```

**Version:**
:   $Revision: 7044 $

**Author:**
:   Len Trigg (trigg@cs.waikato.ac.nz)

**See Also:**: Serialized Form

---

**Field Summary**

**Fields inherited from class weka.experiment.DatabaseUtils**

`BOOL, BYTE, DATE, DOUBLE, EXP_INDEX_TABLE, EXP_RESULT_COL, EXP_RESULT_PREFIX, EXP_SETUP_COL, EXP_TYPE_COL, FLOAT, INTEGER, LONG, PROPERTY_FILE, SHORT, STRING, TEXT, TIME`


**Constructor Summary**

`InstanceQuery()`
  
          Sets up the database drivers


| **Method Summary** | |
| --- | --- |
| `java.lang.String[]` | `getOptions()`             Gets the current settings of InstanceQuery |
| `java.lang.String` | `getQuery()`             Get the query to execute against the database |
| `java.lang.String` | `getRevision()`             Returns the revision string. |
| `boolean` | `getSparseData()`             Gets whether data is to be returned as a set of sparse instances |
| `java.util.Enumeration` | `listOptions()`             Returns an enumeration describing the available options |
| `static void` | `main(java.lang.String[] args)`             Test the class from the command line. |
| `java.lang.String` | `queryTipText()`             Returns the tip text for this property |
| `Instances` | `retrieveInstances()`             Makes a database query using the query set through the -Q option to convert a table into a set of instances |
| `Instances` | `retrieveInstances(java.lang.String query)`             Makes a database query to convert a table into a set of instances |
| `void` | `setOptions(java.lang.String[] options)`             Parses a given list of options. |
| `void` | `setQuery(java.lang.String q)`             Set the query to execute against the database |
| `void` | `setSparseData(boolean s)`             Sets whether data should be encoded as sparse instances |
| `java.lang.String` | `sparseDataTipText()`             Returns the tip text for this property |

**Methods inherited from class weka.experiment.DatabaseUtils**

`arrayToString, close, close, connectToDatabase, createExperimentIndex, createExperimentIndexEntry, createResultsTable, databaseURLTipText, debugTipText, disconnectFromDatabase, execute, experimentIndexExists, getDatabaseURL, getDebug, getKeywords, getKeywordsMaskChar, getPassword, getResultFromTable, getResultSet, getResultsTableName, getSupportedCursorScrollType, getUsername, isConnected, isCursorScrollable, isCursorScrollSensitive, isKeyword, maskKeyword, passwordTipText, processKeyString, putResultInTable, select, setDatabaseURL, setDebug, setKeywords, setKeywordsMaskChar, setPassword, setUsername, tableExists, translateDBColumnType, typeName, update, usernameTipText`

**Methods inherited from class java.lang.Object**

`equals, getClass, hashCode, notify, notifyAll, toString, wait, wait, wait`

**Constructor Detail**

### InstanceQuery

```
public InstanceQuery()
              throws java.lang.Exception
```

:   Sets up the database drivers

    **Throws:**: `java.lang.Exception` - if an error occurs


**Method Detail**

### listOptions

```
public java.util.Enumeration listOptions()
```

:   Returns an enumeration describing the available options

    :   **Specified by:**: `listOptions` in interface `OptionHandler`
    :   **Returns:**: an enumeration of all options

---


### setOptions

```
public void setOptions(java.lang.String[] options)
                throws java.lang.Exception
```

:   Parses a given list of options.
    Valid options are:

    ```
     -Q <query>
      SQL query to execute.
    ```

    ```
     -S
      Return sparse rather than normal instances.
    ```

    ```
     -U <username>
      The username to use for connecting.
    ```

    ```
     -P <password>
      The password to use for connecting.
    ```

    ```
     -D
      Enables debug output.
    ```

    :   **Specified by:**: `setOptions` in interface `OptionHandler`
    :   **Parameters:**: `options` - the list of options as an array of strings **Throws:**: `java.lang.Exception` - if an option is not supported

---


### queryTipText

```
public java.lang.String queryTipText()
```

:   Returns the tip text for this property

    :   **Returns:**: tip text for this property suitable for displaying in the explorer/experimenter gui

---


### setQuery

```
public void setQuery(java.lang.String q)
```

:   Set the query to execute against the database

    :   **Parameters:**: `q` - the query to execute

---


### getQuery

```
public java.lang.String getQuery()
```

:   Get the query to execute against the database

    :   **Returns:**: the query

---


### sparseDataTipText

```
public java.lang.String sparseDataTipText()
```

:   Returns the tip text for this property

    :   **Returns:**: tip text for this property suitable for displaying in the explorer/experimenter gui

---


### setSparseData

```
public void setSparseData(boolean s)
```

:   Sets whether data should be encoded as sparse instances

    :   **Parameters:**: `s` - true if data should be encoded as a set of sparse instances

---


### getSparseData

```
public boolean getSparseData()
```

:   Gets whether data is to be returned as a set of sparse instances

    :   **Returns:**: true if data is to be encoded as sparse instances

---


### getOptions

```
public java.lang.String[] getOptions()
```

:   Gets the current settings of InstanceQuery

    :   **Specified by:**: `getOptions` in interface `OptionHandler`
    :   **Returns:**: an array of strings suitable for passing to setOptions()

---


### retrieveInstances

```
public Instances retrieveInstances()
                            throws java.lang.Exception
```

:   Makes a database query using the query set through the -Q option
    to convert a table into a set of instances

    :   **Returns:**: the instances contained in the result of the query **Throws:**: `java.lang.Exception` - if an error occurs

---


### retrieveInstances

```
public Instances retrieveInstances(java.lang.String query)
                            throws java.lang.Exception
```

:   Makes a database query to convert a table into a set of instances

    :   **Parameters:**: `query` - the query to convert to instances **Returns:**: the instances contained in the result of the query, NULL if the SQL query doesn't return a ResultSet, e.g., DELETE/INSERT/UPDATE **Throws:**: `java.lang.Exception` - if an error occurs

---


### main

```
public static void main(java.lang.String[] args)
```

:   Test the class from the command line. The instance
    query should be specified with -Q sql\_query

    :   **Parameters:**: `args` - contains options for the instance query

---


### getRevision

```
public java.lang.String getRevision()
```

:   Returns the revision string.

    :   **Specified by:**: `getRevision` in interface `RevisionHandler` **Overrides:**: `getRevision` in class `DatabaseUtils`
    :   **Returns:**: the revision


---


|  |  |  |
| --- | --- | --- |
| **Overview** \| **Package** \| **Class** \| **Tree** \| **Deprecated** \| **Index** \| **Help** \| **Weka's home** | |  |
| **PREV CLASS**   **NEXT CLASS** | **FRAMES**    **NO FRAMES**     **All Classes** |  |
| SUMMARY: NESTED \| FIELD \| CONSTR \| METHOD | DETAIL: FIELD \| CONSTR \| METHOD |  |


---
